# Virologic outcomes with tenofovir-lamivudine-dolutegravir in adults failing PI-based second-line ART

**DOI:** 10.4102/sajhivmed.v25i1.1567

**Published:** 2024-04-26

**Authors:** Ying Zhao, Jacqueline Voget, Isaac Singini, Zaayid Omar, Vanessa Mudaly, Andrew Boulle, Gary Maartens, Graeme Meintjes

**Affiliations:** 1 Department of Medicine, Faculty of Health Science, University of Cape Town, Cape Town, South Africa; 2 Wellcome Centre for Infectious Diseases Research in Africa, Institute of Infectious Diseases and Molecular Medicine, University of Cape Town, Cape Town, South Africa; 3 Western Cape Government Department of Health and Wellness, Cape Town, South Africa; 4 Biostatistics Research Unit, South African Medical Research Council, Cape Town, South Africa; 5 Provincial Health Data Centre, Western Cape Department of Health and CIDER, School of Public Health and Family Medicine, University of Cape Town, Cape Town, South Africa; 6 Division of Clinical Pharmacology, Department of Medicine, University of Cape Town, Cape Town, South Africa

**Keywords:** antiretroviral therapy, dolutegravir, HIV, virologic failure, third-line

## Abstract

**Background:**

In South African antiretroviral guidelines, selected patients failing second-line protease inhibitor (PI)-based therapy qualify for genotypic resistance testing – those with PI resistance receive darunavir-based third-line regimens; those without PI resistance continue current regimen with adherence support. The Western Cape province, from September 2020, implemented a strategy of tenofovir-lamivudine-dolutegravir (TLD) for patients, provided there was no tenofovir resistance, irrespective of PI resistance.

**Objectives:**

To evaluate virologic outcomes with TLD among adults failing second-line PI regimens with no tenofovir resistance.

**Method:**

An observational cohort study comparing outcomes in patients switched to TLD with those continuing the same PI or switched to darunavir-based regimens. Follow-up was until virologic suppression (HIV-1 RNA < 400 copies/mL), or at the point of censoring.

**Results:**

One hundred and thirty-three patients switched to TLD, 101 to darunavir-based regimens, and 121 continued with the same PI. By 12 months, among patients with PI resistance, 42/47 (89%) in the TLD group had HIV-1 RNA < 400 copies/mL compared to 91/99 (92%) in the darunavir group (hazard ratio, 1.11; 95% confidence interval, 0.77–1.60). In patients without PI resistance, 66/86 (77%) in the TLD group had HIV-1 RNA < 400 copies/mL compared to 42/120 (35%) in those continuing with the same PI (hazard ratio, 4.03; 95% confidence interval, 2.71–5.98). Two patients receiving TLD developed virologic failure with high-level dolutegravir resistance.

**Conclusion:**

Amongst patients failing second-line PI with no PI resistance, switching to TLD was associated with higher virologic suppression, likely due to improved adherence. Virologic outcomes were similar in patients with PI resistance switched to darunavir-based regimens or TLD.

**What this study adds:** Our study supports switching to TLD as a third-line regimen or an alternative second-line regimen after PI regimen failure in a programmatic setting, with the caveat that patients switched to TLD in our study had no tenofovir resistance.

## Introduction

Nearly 29 million adults are on antiretroviral therapy (ART) globally in 2022, with dolutegravir-based regimens used by 91% of adults in low- to middle-income countries.^1^ Ritonavir-boosted protease inhibitor (PI) regimens have been recommended for two decades as second-line ART in the South African public healthcare sector until recently, with most patients on lopinavir, or atazanavir for those unable to tolerate lopinavir. The proportion of patients with virologic failure on a second-line PI regimen is high in resource-limited settings, reaching 27% by 24 months and 38% by 36 months in a systematic review.^2^

Patients on a second-line PI regimen for over 2 years and failing despite good adherence qualify for genotypic antiretroviral resistance testing (GART) to decide the need for and choice of a third-line regimen in the South African public sector programme.^3^ This policy is based on evidence that higher rates of adherence and longer exposure to PI regimens predict resistance to PI.^4^ If major PI mutations are detected, patients are switched to a third-line regimen that includes ritonavir-boosted darunavir (DRV/r), together with nucleoside reverse transcriptase inhibitors (NRTIs)_ the regimen may include dolutegravir and/or etravirine directed by GART.^5^ Most patients failing a second-line PI regimen have no major PI mutations,^6^ and the same second-line regimen is continued with intensified adherence support, as the absence of PI resistance implies that poor adherence is the cause of failure. The guidelines do allow for switching from lopinavir to atazanavir when there are gastro-intestinal side effects. The 2023 update to the Southern African HIV Clinicians Society guidelines recommend that patients who experience virologic failure on a second-line PI regimen can be switched to tenofovir-lamivudine-dolutegravir (TLD), provided darunavir is reported as fully susceptible and there has been no prior dolutegravir failure.^7^

In the Western Cape province, from September 2020, an adaption to the national guideline allowed switching to TLD in patients failing a second-line PI regimen, provided that tenofovir was fully active on GART (i.e. Stanford score < 10). This was done regardless of whether the GART demonstrated susceptibility or resistance to the PI the patient was taking. Studies had shown that combining dolutegravir with one fully active NRTI was sufficient to achieve a high proportion of virologic suppression in both first- and second-line regimens.^8,9^ Favourable tolerability profile and low pill burden make the fixed-dose combination of the TLD regimen a desirable treatment option for this vulnerable group of patients who struggle with adherence and engagement with care. No data exist on the virologic outcomes of TLD as a third-line regimen or an alternative second-line regimen after second-line PI regimen failure. Among patients failing a PI (lopinavir or atazanavir) second-line regimen in whom GART was performed, we conducted an observational cohort study to compare outcomes in patients switched to TLD with those continuing the same PI (when no PI resistance was detected) and compared outcomes in patients switched TLD with those switched to DRV/r-based regimens (when PI resistance was detected).

## Research methods and design

### Study population and eligibility criteria

Access to GART and third-line ART is managed centrally by the HIV/AIDS STI and TB (HAST) Directorate at the Western Cape Provincial Department of Health. The Provincial Third Line Committee includes HIV expert clinicians and virologists, who advise on the appropriate choice of a third-line regimen, continuation of the second-line regimen, or switch to an alternative second-line regimen after reviewing treatment history and GART. We screened all applications to the Western Cape Provincial Third Line Committee and included consecutive patients with virologic failure who had been on a ritonavir-boosted lopinavir or atazanavir second-line regimen for at least 2 years, and in whom a GART was performed. Virologic failure was defined as at least two HIV-1 RNA ≥ 1000 copies/mL despite adherence optimisation.^3^ Exclusion criteria were: no dispensing data after GART, prior exposure to an integrase inhibitor, switching to zidovudine-lamivudine-dolutegravir, and < 18 years old. Commencement of ART was checked against the pharmacy claims history on the electronic Provincial Single Patient Viewer (SPV) system and patients who had > 6 months delay in initiating a new regimen or dispensing of the same PI regimen after GART were excluded.

### Procedures

Samples were collected for GART prior to initiation of any third-line regimen. GART was performed at the National Health Laboratory Service (NHLS) Virology Laboratory at Tygerberg Hospital in Cape Town, South Africa, and drug-susceptibility prediction was performed with the Stanford algorithm (version 8.9).^10^ Following a switch to third-line regimens (TLD or DRV/r-based) or a decision to remain on the same PI regimen, patients were followed up and monitored at their local clinics. A REDCap electronic database registry on the University of Cape Town server was designed to record outcomes of patients failing second-line PI regimens in the Western Cape province, and clinical data from the application forms submitted to the Provincial Third Line Committee, the Provincial SPV, and NHLS records were entered into the database.

### Outcome measures

The primary outcome was time to virologic suppression (defined as HIV-1 RNA < 400 copies/mL). First, comparing those switched to TLD after second-line PI regimen failure with those continuing the same PI regimen (when no resistance to PI was detected at the time of second-line failure) and, second, comparing those switched to TLD with those switched to a DRV/r-based regimen (when resistance to PI was detected at the time of second-line failure). PI resistance was defined as a Stanford score ≥ 10, indicating at least potential low-level resistance. Patients were followed up until the primary outcome, or censored at death, loss to follow-up, switching ART drugs (i.e. switching from PI to dolutegravir or vice versa), or date of administrative censure (27 July 2023). Patients were considered lost to follow-up when there was a gap of > 3 months in clinic visits or dispensing of ART after the last recorded healthcare contact and no further contact by 12 months.

The secondary outcomes included time to HIV-1 RNA < 50 copies/mL, all-cause mortality at 12 months, proportions with virologic suppression at 6 months and 12 months (with a window of ±3 months), and emergence of integrase and new NRTI resistance mutations in those who experienced virologic failure on TLD (defined as two consecutive HIV-1 RNA ≥ 1000 copies/mL after at least 3 months on the TLD regimen).

### Statistical considerations

Time-to-event outcomes were analysed using Kaplan-Meier (KM) survival analysis and compared by group using the log-rank test. For patients switched to TLD or DRV/r-based regimens, the date of starting the new regimen was used as the entry point. For patients who continued the same PI regimen, the date of first dispensing of the PI regimen after GART was used as the entry point. Cox proportional hazards models (for patients with and without resistance to PI at the time of second-line failure) were developed to analyse predictors of suppression using Stata software, version 17.0 (StataCorp), with all variables hypothesised to predict virologic suppression (to < 400 copies/mL and < 50 copies/mL) included in the models.

### Ethical considerations

This study was approved by the Human Research Ethics Committee (HREC) of the University of Cape Town (reference 104/2022). As routine clinical data were collected in the database registry and used for observational research, which carried minimal risk to patients, a waiver for the requirement of informed consent was granted with the registry approved by the HREC at the University of Cape Town (reference R013/2021). The database was secured as a password-protected REDCap electronic registry on the University of Cape Town server. Patient identifiers were removed from the datasets for analysis.

## Results

**FIGURE 1:**
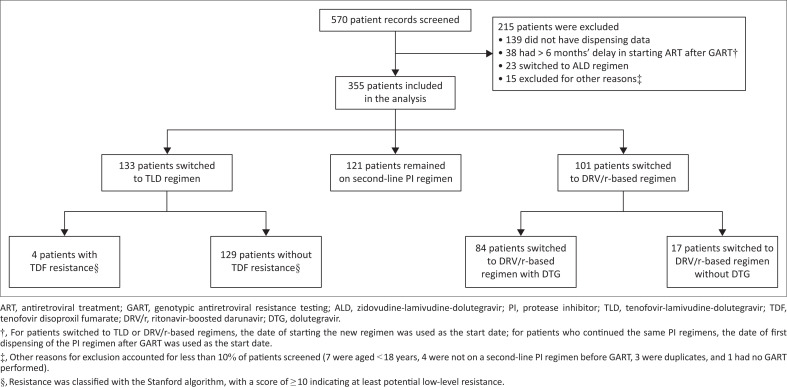
Flow diagram showing screening and inclusion of the study population.

We screened 570 applications for GART received by the Western Cape Provincial Third Line Committee between August 2019 and November 2021. Data from 355 patients with virologic failure on a second-line PI regimen were analysed; these included 148 patients with PI resistance and 207 patients without PI resistance (Figure 1). Among the 355 patients, the proportion with at least low-level resistance to lopinavir was 36%, and to atazanavir, 38%. Of those with PI resistance, 47 switched to TLD and 101 switched to DRV/r-based regimens (84% switched to DRV/r with dolutegravir and other drugs). Intermediate-level resistance or high-level resistance to darunavir was present in 12.8% in the TLD group and 13.1% in the DRV/r group (Online Appendix 1, Table S1). Of those without PI resistance, 86 switched to TLD and 121 continued the same PI regimen.

Demographics and clinical characteristics at the time of GART are summarised in Table 1. Of the 133 patients in the TLD group, 4 (3%) had mutations associated with at least low-level resistance to tenofovir at the time of second-line failure; these four patients were included in the analyses. Details of the resistance pattern and outcomes of these four patients are shown in Online Appendix 1, Table S2. A small proportion (2%) in the TLD group switched before September 2020. The groups were well matched other than for proportions with tenofovir exposure at first-line ART failure, which was higher in the TLD group (82%) among patients with PI resistance, and duration of ART exposure, which was shorter in the TLD group. Two patients who had PI resistance on GART continued the same PI regimens and one patient who had no PI resistance was switched to a DRV/r-based regimen; these three patients were excluded from the KM analyses.

**TABLE 1:** Baseline demographic and clinical characteristics of patients.

Variable	PI resistance†									No PI resistance†								
	TLD group (*n* = 47)				DRV/r group (*n* = 99)				*P* ‡	TLD group (*n* = 86)				Continue PI group (*n* = 120)				*P* ‡
	*n*	%	Median	IQR	*n*	%	Median	IQR		*n*	%	Median	IQR	*n*	%	Median	IQR	
Age, years	-	-	42	36–48	-	-	40	36–47	0.521	-	-	40	34–46	-	-	40	33–46	0.636
Female gender	31	66.0	-	-	63	63.6	-	-	0.784	60	69.8	-	-	90	75	-	-	0.405
Weight, kg	-	-	74	65–85	-	-	70	59-83	0.125	-	-	64	57–73	-	-	62	55–71	0.205
CD4+ cell count, cells/μL§	-	-	189	84–297	-	-	170	53–322	0.971	-	-	137	96–230	-	-	135	71–227	0.568
HIV-1 RNA, log_10_ copies/mL§	-	-	4.4	3.6–5.0	-	-	4.7	4.1–4.9	0.122	-	-	4.8	4.1–5.4	-	-	4.7	4.3–5.2	0.977
Duration of ART exposure, years	-	-	7.0	6.0–11.0	-	-	10.0	7.0–13.0	0.004	-	-	9.0	6.0–11.0	-	-	8.0	5.8–11.0	0.204
Duration of PI exposure, years	-	-	5.0	2.5–7.5	-	-	6.0	4.0–7.0	0.237	-	-	4.0	2.0–7.0	-	-	4.0	2.0–7.0	0.881
Receiving tenofovir at the time of first-line failure	36¶	81.8¶	-	-	54††	55.7††	-	-	0.003	55‡‡	68.8‡‡	-	-	77§§	65.8§§	-	-	0.667
Entry date before September 2020¶¶	1	2.1	-	-	52	52.5	-	-	< 0.001	1	1.2	-	-	58	48.3	-	-	< 0.001
**Genotypic antiretroviral resistance testing**																		
Lopinavir resistance†	45	95.7	-	-	93	93.9	-	-	0.712	0	0.0	-	-	0	0.0	-	-	1.000
Atazanavir resistance†	47	100.0	-	-	99	100.0	-	-	1.000	0	0.0	-	-	0	0.0	-	-	1.000
Darunavir resistance†	23	48.9	-	-	45	45.5	-	-	0.831	0	0.0	-	-	0	0.0	-	-	1.000
**Major PI mutations** †††									1.000									1.000
0	1	2.1	-	-	3	3.0	-	-	-	85	98.8	-	-	118	98.3	-	-	-
1–2	17	36.2	-	-	37	37.4	-	-	-	1	1.2	-	-	2	1.7	-	-	-
≥ 3	29	61.7	-	-	59	59.6	-	-	-	0	0.0	-	-	0	0.0	-	-	-
Tenofovir resistance†	1	2.1	-	-	69	69.7	-	-	< 0.001	3	3.5			11	9.2			0.110

PI, protease inhibitor; TLD, tenofovir-lamivudine-dolutegravir; DRV/r, ritonavir-boosted darunavir; IQR, interquartile range; ART, antiretroviral therapy.

† , Resistance was classified with the Stanford algorithm, with a score of ≥ 10 indicating at least potential low-level resistance.

‡ , *P* values calculated using Wilcoxon rank sum test for continuous variables and χ^2^ test for binary variables.

§ , The data for CD4 + cell count and HIV-1 RNA were recorded at the time of second-line PI regimen failure.

¶ , Denominator: *n* = 44.

†† , Denominator: *n* = 97.

‡‡ , Denominator: *n* = 80.

§§ , Denominator: *n* = 117.

¶¶ , For those switched to TLD or DRV/r-based regimen, the date of starting the new regimen was used as the entry date. For those continued with the same PI regimen, the date of first dispensing of the PI regimen after GART was used as the entry date.

††† , Major PI mutations were defined as: I47A/V, I50I/L/V, I54A/I/V/T/M, I84I/V, L76L/V, L90L/M, M46I/M/L, N88N/S, V32I/V, V82A/V/C/I/L/M, and G48A/V.

**FIGURE 2:**
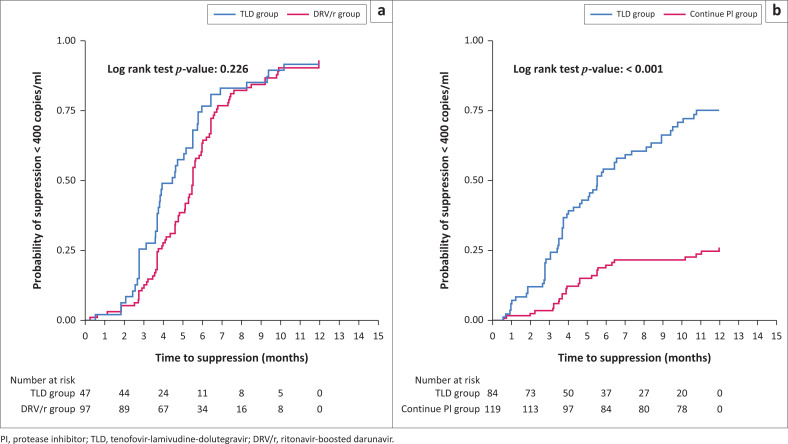
Kaplan-Meier graph for time to virological suppression (HIV-1 RNA < 400 copies/mL) during the first 12 months of therapy; (a) compares the tenofovir-lamivudine-dolutegravir (TLD) and ritonavir-boosted darunavir (DRV/r) groups in patients with protease inhibitor (PI) resistance, (b) compares TLD and continue same PI groups in patients without PI resistance.

Time to HIV-1 RNA < 400 copies/mL by study arm is shown in Figure 2. In patients with PI resistance, 42 (89%; 95% confidence interval [CI], 77% – 96%) of 47 patients in the TLD group achieved HIV-1 RNA < 400 copies/mL by 12 months compared to 91 (92%; 95% CI, 85% – 96%) of 99 patients in the DRV/r group; the crude hazard ratio (HR) for virologic suppression in the TLD group was 1.11 (95% CI, 0.77–1.60; *P* = 0.562; Figure 2a). In patients without PI resistance, 66 (77%; 95% CI, 66% – 85%) of 86 patients in the TLD group achieved HIV-1 RNA < 400 copies/mL by 12 months compared to 42 (35%; 95% CI, 27% – 44%) of 120 patients in the continue PI group; the crude HR for virologic suppression in the TLD group was 4.03 (95% CI, 2.71–5.98; *P* < 0.001; Figure 2b) – this effect persisted after adjustment for HIV-1 RNA and CD4+ cell count at the time of second-line failure in a multivariate Cox proportional hazard model (adjusted HR, 3.96; 95% CI, 2.25–6.98; *P* < 0.001).

Factors associated with time to HIV-1 RNA < 400 copies/mL in patients without PI resistance are shown in Table 2. In univariate analysis, higher HIV-1 RNA at the time of second-line failure was associated with failure to achieve HIV-1 RNA < 400 copies/mL (unadjusted HR for every log_10_ increase in HIV-1 RNA, 0.91; 95% CI, 0.84–0.98) – this remained an independent predictor after adjustment for treatment group (adjusted HR, 0.86; 95% CI, 0.74–0.99). Factors associated with time to HIV-1 RNA < 400 copies/mL in patients with PI resistance are shown in Table 3. Having received tenofovir at the time of first-line failure, which may result in archived or minority population tenofovir-resistant variants, was not associated with increased risk of virologic failure (adjusted HR, 1.05; 95% CI, 0.66–1.67 for those with PI resistance, and adjusted HR, 0.94; 95% CI, 0.53–1.65, for those without PI resistance).

**TABLE 2:** Predictors of virologic suppression (HIV-1 RNA < 400 copies/mL) by 12 months in patients without protease inhibitor resistance.

Variable	Univariate HR	95% CI	*P*	Multivariate HR	95% CI	*P*
Switched to TLD	4.03	2.71–5.98	< 0.001	3.96	2.25–6.98	< 0.001
Male gender	1.25	0.95–1.63	0.110	1.03	0.60–1.79	0.909
Receiving tenofovir at the time of first-line failure	1.08	0.82–1.41	0.590	0.94	0.53–1.65	0.824
Age (per year)	0.99	0.98–1.01	0.352	1.00	0.97–1.02	0.723
Duration of PI exposure (per year)	1.00	0.97–1.05	0.809	1.00	0.90–1.10	0.921
CD4+ cell count at the time of second-line failure (per square root cells/μL)	1.02	0.99–1.05	0.294	1.02	0.97–1.07	0.505
HIV-1 RNA at the time of second-line failure (per log_10_ copies/mL)	0.91	0.84–0.98	0.010	0.86	0.74–0.99	0.040

PI, protease inhibitor; HR, hazard ratio; CI, confidence interval; TLD, tenofovir-lamivudine-dolutegravir.

**TABLE 3:** Predictors of virologic suppression (HIV-1 RNA < 400 copies/mL) by 12 months in patients with protease inhibitor resistance.

Variable	Univariate HR	95% CI	*P*	Multivariate HR	95% CI	*P*
Switched to TLD	1.11	0.77–1.60	0.562	0.82	0.53–1.28	0.379
Male gender	1.25	0.95–1.63	0.110	1.26	0.77–2.07	0.356
Receiving tenofovir at the time of first-line failure	1.08	0.82–1.41	0.590	1.05	0.66–1.67	0.847
Age (per year)	0.99	0.98–1.01	0.352	0.99	0.96–1.01	0.286
Duration of PI exposure (per year)	1.00	0.97–1.05	0.809	1.07	0.98–1.16	0.127
CD4+ cell count at the time of second-line failure (per square root cells/μL)	1.02	0.99–1.05	0.294	1.01	0.97–1.05	0.685
HIV-1 RNA at the time of second-line failure (per log_10_ copies/mL)	0.91	0.84–0.98	0.010	0.90	0.79–1.02	0.093

PI, protease inhibitor; HR, hazard ratio; CI, confidence interval; TLD, tenofovir-lamivudine-dolutegravir.

**FIGURE 3:**
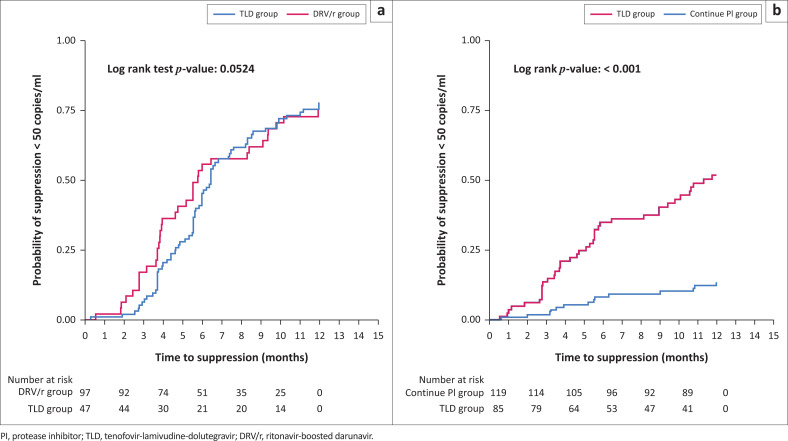
Kaplan-Meier graph for time to virological suppression (HIV-1 RNA < 50 copies/mL) during the first 12 months of therapy; (a) compares tenofovir-lamivudine-dolutegravir (TLD) and ritonavir-boosted darunavir (DRV/r) groups in patients with protease inhibitor (PI) resistance, (b) compares TLD and continue same PI groups in patients without PI resistance.

Time to HIV-1 RNA < 50 copies/mL by study arm is shown in Figure 3. The KM estimates of cumulative proportion of HIV-1 RNA < 50 copies/mL by 12 months among patients with PI resistance in the TLD group were 70%, and 75% in the DRV/r group (unadjusted HR, 0.88; 95% CI, 0.60–1.30; *P* = 0.528). In patients without PI resistance, 52% in the TLD group achieved HIV-1 RNA < 50 copies/mL by 12 months compared to 26% in the continue PI group (unadjusted HR, 4.95; 95% CI, 2.97–8.24; *P* < 0.001). Factors associated with HIV-1 RNA < 50 copies/mL are shown in Online Appendix 1, Tables S3 and S4. In multivariate analysis among patients with PI resistance, we found those with longer exposure to PI regimen at the time of second-line failure were more likely to suppress (adjusted HR for every year increase in PI exposure, 1.11; 95% CI, 1.02–1.21), whereas patients with higher HIV-1 RNA at second-line ART failure were less likely to suppress (adjusted HR for every log_10_ increase in HIV-1 RNA, 0.83; 95% CI, 0.72–0.96). Virologic outcomes by study arm at 6 months and 12 months are shown in Online Appendix 1, Table S5. In patients with PI resistance, death occurred in 0/46 (0.0%) in the TLD group and 4/94 (4.3%) in the DRV/r group by 12 months. In patients without PI resistance, death occurred in 3/77 (3.9%) in the TLD group and 11/110 (10.0%) in the continued PI group by 12 months.

Two (2%) patients in the TLD group were known to have developed virologic failure with dolutegravir resistance. One patient developed virologic rebound with high-level dolutegravir resistance (E138K, G140A, and Q148K mutations) at month 10 after achieving an HIV-1 RNA < 400 copies/mL at month 2; GART at the time of second-line failure detected resistance to tenofovir and lamivudine (M184V, T215Y, and M41L/M mutations). One patient had HIV-1 RNA > 1000 copies/mL from month 4 to month 31 and a repeat GART detected high-level dolutegravir resistance (E138K, E157Q, G118R, and T66A mutations) at month 31; tenofovir was fully active on GART at switch. After detection of dolutegravir resistance, both patients switched to DRV/r-based regimens as recommended by the Provincial Third Line Committee. Of the 121 patients in the continue PI group, four switched to TLD by 12 months due to prolonged virologic failure and without a repeat GART.

## Discussion

Our results provide new information relevant for clinical practice and policy in this ART-experienced population with virologic failure on a second-line PI regimen. Switching to TLD when there was no PI resistance was associated with higher virologic suppression compared with those who continued the same PI regimens – a finding that suggests improved adherence in those switched to the TLD regimen. Virologic suppression was comparable in patients with PI resistance switched to a DRV/r-based regimen or TLD. Our findings strengthen the evidence base for switching to TLD as a third-line regimen or an alternative second-line regimen after PI regimen failure, provided there is no tenofovir resistance, in programmatic settings.

The virologic outcomes observed in the DRV/r group of our study are consistent with those from other observational studies (two of which were conducted in South Africa) assessing the efficacy of third-line ART in clinical practice.^11,12,13^ In our study, the majority of patients (65%) who continued the same failing PI regimen with adherence support failed to re-suppress their HIV-1 RNA to ≤ 400 copies/mL by 12 months. By contrast, a study among patients failing second-line regimens reported 64% suppression following intensified adherence support.^14^ Poor tolerability and high pill burden make PI regimens more difficult to adhere to than non-PI regimens, and prolonged virologic failure may result in accumulation of PI mutations.^15,16^

In our study, the proportion of patients achieving virologic suppression to < 400 copies/mL with the TLD regimen among those with PI resistance was similar to that achieved in the Second-Line Switch to Dolutegravir (2SD) trial conducted in Kenya in which patients with virologic suppression on PI regimens were switched to dolutegravir with two NRTIs or continued with their PI regimens.^17^ Our results are striking given that this cohort of patients had previously failed multiple lines of ART, including a PI regimen. The efficacy of the TLD regimen after failure of first-line non-nucleoside reverse transcriptase inhibitors (NNRTI)-based regimens is well-established.^18,19,20^ Our results suggest that TLD achieves a high proportion of virologic suppression after failure of second-line PI regimens when tenofovir is fully active on GART. Amongst patients in the TLD groups, those without PI resistance were less likely to achieve virologic suppression compared to those with PI resistance, likely explained by adherence differences. Studies have shown that patients who had suboptimal adherence to a first-line regimen were more likely to have suboptimal adherence to subsequent lines of ART.^21^ Patients with no PI mutations on GART despite virologic failure on a second-line PI regimen (which implies a lack of adherence with the PI regimen) who are switched to TLD should be targeted for intensified adherence support.

Because most patients were not receiving tenofovir at the time of second-line failure when GART was performed, prior K65R mutation (selected for by tenofovir and conferring intermediate resistance to tenofovir) as a consequence of tenofovir-containing first-line regimen failure may have been archived in viral reservoirs or circulating at low concentrations under the limit of detection for conventional genotyping (< 20%). Minority variants with resistance to NNRTIs were associated with an increased risk of virologic failure on a first-line NNRTI regimen in a systematic review.^22^ There has been concern that archived or minority population tenofovir-resistant variants could be selected for proliferation under drug pressure with the TLD regimen and compromise tenofovir activity, resulting in dolutegravir being used with two NRTIs to which there is resistance, in turn risking the development of dolutegravir resistance and treatment failure. Most of our patients in the TLD group received tenofovir at the time of first-line failure and this prior tenofovir exposure was not associated with increased risk of virologic non-suppression ≥ 400 copies/mL. Minority K65R variants at low concentrations were not selected for proliferation under drug pressure within a PI regimen, which has a higher genetic barrier to resistance than an NNRTI regimen.^23^ Our findings do not suggest that these minority variants drive treatment failure with a dolutegravir-based regimen.

We observed a low incidence of treatment-emergent dolutegravir resistance (2% of all receiving TLD) among patients who were known to have developed virologic failure at 1–3 years after switching to TLD. Emergent dolutegravir resistance has been reported infrequently (1% – 4%) in second-line ART.^24,25^ In a paediatric population aged 12–18 who were PI regimen experienced and receiving weight-based dolutegravir, eight cases of dolutegravir resistance were detected in 142 patients included in the analysis.^26^ Resistance to NRTIs is associated with a substantial increase in the risk of dolutegravir resistance (adjusted odds ratio 5.2 in the presence of potential low-level or low-level NRTI resistance, and 13.4 in the presence of intermediate-level or high-level NRTI resistance, respectively).^27^ Our findings highlight the need for awareness of the higher risk of dolutegravir resistance in patients with prior regimen failure compared to those never having failed a regimen (i.e. have fully active NRTIs). Appropriate management algorithms to ensure resistance is detected timeously, as well as objective measures of adherence to avoid unnecessary resistance tests, should be part of surveillance initiatives with the current transition to dolutegravir-based regimens for treatment-experienced patients.

Our study has limitations. First, we included patients with treatment failure on second-line PI regimens as of August 2019 and switching to TLD in such patients was implemented and phased in rapidly from September 2020, which may have introduced biases related to temporal effects. The majority (98%) of patients in the TLD group switched after September 2020, compared with 47% in the DRV/r group and 52% in the continue PI group. Second, the relatively small sample size in patients with resistance to PI switching to TLD or DRV/r-based regimens limited the precision of our primary endpoint estimates in those groups. Third, of those switched to DRV/r-based regimens, the majority (84%) switched to DRV/r with dolutegravir and other drugs. Our findings may not be generalisable to patients switching from second-line PI regimens to only DRV/r with two NRTIs. Fourth, most of our patients (97%) in the TLD group had fully active tenofovir on GART at second-line ART failure. Our findings may not be generalisable to viraemic patients switching from second-line PI regimens to TLD with resistance to both tenofovir and lamivudine at the time of second-line failure. While there are no randomised controlled trials assessing the efficacy of TLD as a third-line regimen after virologic failure on second-line PI regimens, switching to TLD could be effective in most patients even if resistance to both tenofovir and lamivudine is present, based on recent evidence showing that recycling tenofovir in second-line ART achieves acceptable rates of virologic suppression.^18,19,20^ Fifth, some potential predictors of virologic outcomes, such as measures of adherence or drug-drug interactions, were not assessed in our study.

## Conclusion

Amongst patients who had virologic failure on second-line PI regimens, a high proportion of those with PI resistance switched to TLD or DRV/r-based regimens achieved virologic suppression. The improved virologic outcomes with switching from a failing PI regimen to TLD when there was no PI resistance in comparison to remaining on the same PI regimen was likely driven by improved adherence with a better tolerated single-tablet regimen. Our results provide evidence to support switching to TLD after PI regimen failure with the caveat that only patients without tenofovir resistance were switched to TLD in this study.
